# Mismatch Repair Deficiency and Microsatellite Instability in Triple-Negative Breast Cancer: A Retrospective Study of 440 Patients

**DOI:** 10.3389/fonc.2021.570623

**Published:** 2021-03-04

**Authors:** Xin-yu Ren, Yu Song, Jing Wang, Long-yun Chen, Jun-yi Pang, Liang-rui Zhou, Song-jie Shen, Xi Cao, Yu-xin Wang, Miao-miao Shao, Zhi-yong Liang, Qiang Sun, Huan-wen Wu

**Affiliations:** ^1^ Department of Pathology, State Key Laboratory of Complex Severe and Rare Diseases, Molecular Pathology Research Center, Peking Union Medical College Hospital, Chinese Academy of Medical Science and Peking Union Medical College, Beijing, China; ^2^ Department of Breast Surgery, Peking Union Medical College Hospital, Chinese Academy of Medical Science and Peking Union Medical College, Beijing, China; ^3^ Beijing Hospital of Traditional Chinese Medicine Affiliated Pinggu Hospital, Beijing, China; ^4^ Research and Development Department (R&D), Beijing Microread Genetics Co., Ltd., Beijing, China

**Keywords:** triple-negative breast cancer (TNBC), mismatch repair (MMR) deficiency, microsatellite instability, prognosis, mismatch repair proficiency

## Abstract

**Purpose:**

To investigate the status of mismatch repair (MMR) and microsatellite instability (MSI) in triple-negative breast cancer (TNBC) and to examine correlations between MMR/MSI status and clinicopathological parameters.

**Methods:**

We retrospectively collected tissue samples from 440 patients with TNBC and constructed tissue microarrays. Protein expression of MLH1, MSH2, MSH6, and PMS2 was detected by immunohistochemistry (IHC). We also analyzed 195 patient samples using MSI polymerase chain reaction (PCR) testing. Correlations between MSI status and clinicopathological parameters and prognosis were analyzed.

**Results:**

The median age of the cohort was 49 years (range: 24–90 years) with a median follow-up period of 68 months (range: 1–170 months). All samples were positive for MLH1, MSH2, MSH6, and PMS2, except for one sample identified as MMR-deficient (dMMR) by IHC, with loss of MSH2 and intact MSH6 expression. MSI PCR revealed no case with high-frequency MSI (MSI-H), whereas 14 (7.2%) and 181 (92.8%) samples demonstrated low-frequency and absence of MSI events, respectively. The dMMR sample harbored low-frequency instability, as revealed by MSI PCR, and a possible *EPCAM* deletion in the tumor, as observed from next-generation sequencing. No correlations were detected between MMR or MSI status and clinicopathological parameters, programmed cell death 1 (PD-1)/programmed cell death ligand 1 (PD-L1) expression, or survival.

**Conclusions:**

The incidence of dMMR/MSI-H is extremely low in TNBC, and rare discordant MSI PCR/MMR IHC results may be encountered. Moreover, MMR/MSI status may be of limited prognostic value. Further studies are warranted to explore other predictive immunotherapy biomarkers for TNBC.

## Introduction

Triple-negative breast cancer (TNBC) is characterized by poor prognosis and lack of effective targeted therapies. Most TNBCs are rich in tumor-infiltrating lymphocytes (TILs), the presence of which correlates with tumor immune response activation and sensitivity to chemotherapy, suggestive of better overall survival (1). Immunotherapy has become an indispensable treatment strategy for cancer, as it has shown effective results across several solid tumors. Inhibition of the T-cell inhibitory molecule programmed cell death ligand 1 (PD-L1) has been proven clinically effective in the treatment of multiple cancers (2). In breast cancer, PD-L1 levels are correlated with TIL levels and the complete response to neoadjuvant chemotherapy. Therefore, PD-L1 can be used as a biomarker to identify patients who could benefit from immunotherapy (3). However, no efficient immunotherapy biomarkers are known for TNBC. PD-L1 was initially regarded as an efficient biomarker to predict the efficacy of immunotherapy, however, our previous study revealed that its expression is very low in TNBC tumors, specifically in Chinese women (4). Thus, it is important to identify novel immunotherapy biomarkers for TNBC.

Microsatellite instability (MSI) is caused by defective DNA mismatch repair (dMMR) genes and is characterized by a decrease or increase in repeated nucleotide sequences, which can lead to evasion of apoptosis, development of malignant mutations, and tumorigenesis (5, 6). MSI is a marker of dMMR. MSI status can be determined by immunohistochemistry (IHC), polymerase chain reaction (PCR), and next-generation sequencing (NGS) (6). Generally, the PCR and NGS methods divide tumors into high-frequency MSI (MSI-H), low-frequency MSI (MSI-L), or microsatellite stable (MSS) tumors (7). dMMR and MSI-H have been found in various tumors, such as uterine, ovarian, colorectal, small intestinal, stomach, urothelial, central nervous system, and adrenal gland tumors (8, 9). Germline pathogenic mutations in the DNA mismatch repair genes are also the hallmarks of Lynch syndrome, an autosomal dominant disorder associated with a genetic predisposition for developing a wide spectrum of cancers. Both dMMR and MSI-H have been demonstrated as effective predictors of immunotherapy response (10, 11), and have been approved by the United States Food and Drug Administration as biomarkers for the treatment of solid tumors with immune checkpoint inhibitors targeting programmed death 1 (PD-1), regardless of tumor origin (12).

dMMR/MSI-H have also been evaluated as potential prognosticators and therapeutic targets in several cancers. However, the prognostic value of these biomarkers varies between tumor types. For example, dMMR/MSI-H has been associated with poor prognosis in individuals with colorectal cancer that was insensitive to 5-fluorouracil (FU)-based adjuvant chemotherapy (13), while dMMR/MSI-H were associated with good prognosis in gastric cancer (14). For this reason, MMR/MSI status has been screened in a variety of tumors. Approximately 15% of colon tumors and 15–31% of uterine tumors are MSI-H (15, 16). For hepatocellular carcinoma in non-alcoholic and non-virally infected livers, 16% of tumors (from 37 patients) were MSI-H (17). In a meta-analysis of 48 studies describing 18,612 patients with gastric cancer, MSI-H was observed in 9.2% of patients (14). The prevalence of dMMR/MSI-H in pancreatic ductal adenocarcinoma (PDAC) varied greatly between different subject cohorts, ranging from 0% to 22% (18, 19).

However, data on the prevalence and the prognostic significance of dMMR/MSI-H in breast cancer is limited, especially for TNBC. Although there have been studies on MMR/MSI status in breast cancer, the number of cases is often small, with the largest cohort comprising 444 patients, only 23 of which were TNBC (20). The proportion of MSI-H in these groups varied largely, from 0.2% to 18.6% (20, 21). Most of the available studies used only a single method to evaluate the MMR/MSI status. Furthermore, estrogen receptor (ER)-positive and ER-negative cases were mixed. Several studies have shown that dMMR/MSI-H correlates with resistance to chemotherapy and poor prognosis, while other studies reported that patients with dMMR/MSI-H lived longer than ER-negative breast cancer patients treated with chemotherapy (20, 22–24). Therefore, further verification of the relationship between MMR/MSI status and prognosis is needed. In this study, we enrolled 440 patients with TNBC to investigate MMR/MSI status at both protein and nucleic acid level. We further evaluated the prognostic role of MMR/MSI status and its potential correlations with clinicopathological features, including PD1/PD-L1 expression.

## Materials and Methods

### Patient Samples and Follow-Up Information

440 patients with unilateral TNBC undergoing breast surgery were enrolled, and their FFPE tissues were collected. All patients had been diagnosed at Peking Union Medical College Hospital from May, 2002 to December, 2014, and received standard treatments according to established protocols, including curative surgery, chemotherapy, and radiotherapy. Primary Tumor/Regional Lymph Nodes/Distant Metastasis (TNM) stage was classified according to the AJCC 8th edition. Those treated with neoadjuvant chemotherapy, and those who provided insufficient tissue samples were excluded. Patients with stage IV breast cancer either received neoadjuvant therapy or provided biopsy specimens only, thus there were no stage IV cases in our cohort. Two pathologists independently evaluated the appropriate tumor sections. 195 consecutive cases between May, 2002 and December, 2010 were subjected to both PCR and immunohistochemistry analyses. The rest of consecutive cases between January, 2011 and December, 2014 in the cohort were subjected to IHC analysis only. Patients with MSI-H or dMMR status were further subjected to next-generation sequencing to identify the cause of their defective mismatch repair mechanism.

The follow-up period for this retrospective study was from the date of surgery to March 31st, 2019. Overall survival (OS) was defined as the time from diagnosis to the time of death due to any reason. Disease-free survival (DFS) was defined as the time from diagnosis to the first relapse of the disease (local, regional, or distant metastasis, or contralateral breast cancer). Disease recurrence and metastases were confirmed by diagnostic imaging and pathology.

### Ethical Approval

This study was approved by Peking Union Medical College Hospital Institutional Review Board (PUMCH IRB). All procedures performed in this study involving human participants were in accordance with the ethical standards of the institutional and national research committee, as well as the Declaration of Helsinki and its later amendments. Informed consent of written form was obtained from all individual participants included in the study.

### Tissue Microarray Preparation and Pathological Analysis

A Quick-Ray Manual Tissue Microarrayer (UT-06, UNITMA) was used to construct the tissue microarrays. Three 1-mm cores per case were obtained with a needle and arrayed in a recipient block. Two pathologists assessed the pathological parameters of each sample, including histological differentiation, lympho-vascular invasion, and TILs. TILs evaluation was carried out according to the methods described previously (25). Pathological staging was performed according to the 8^th^ edition of the American Joint Committee on Cancer’s TNM staging system (26).

### Immunohistochemistry

The expression of MMR proteins MLH1, MSH2, MSH6, and PMS2 was detected by immunohistochemistry on an FFPE tissue microarray using Ventana Benchmark XT autostainer (Ventana Medical Systems Inc., Tucson, AZ) according to the manufacturer’s protocols. Antibody information and respective optimizations are listed in **Table 1**. Absence of nuclear staining in tumor cells was considered “loss of expression” with intervening stromal positivity serving as an internal control. ER, PR, and HER2 status were assessed based on the ASCO/CAP guidelines (27). TNBC was defined as HER2 negativity and ER and PR nuclear staining in <1% of the tumor cells. HER2 negativity was determined by either negative (0 or 1) or equivocal (2+) HER2 staining by IHC and no *HER2* gene amplification revealed by fluorescence *in situ* hybridization. The expression of ER, PR, HER2, P53, proliferation marker Ki-67 and basal-like markers (cytokeratin 5/6, extracellular growth factor receptor/EGFR, cytokeratin 14), was detected at the time of diagnosis. Basal-like phenotype of TNBC was defined by positivity for any of the three basal-like markers in the present study. PD-L1 and PD-1 were evaluated as previously described (4).

**Table 1:** Antibody and IHC information for MLH1, MSH2, MSH6, and PMS2.

Antibody	Clone	Dilution	Source	Positive style		Antigen Retrieval*		Incubation
MLH1	Mouse Monoclonal (M1)	Prediluted	Ventana		Nuclear staining	95°C, 88min		36°C, 16min
PMS2	Mouse Monoclonal (A16-4)	Prediluted	Ventana		Nuclear staining	95°C, 92min		36°C, 6min
MSH2	Rabbit Monoclonal (G219-1129)	Prediluted	Ventana		Nuclear staining	95°C, 88min		36°C, 32min
MSH6	Rabbit Monoclonal (SP93)	Prediluted	Ventana		Nuclear staining	95°, 88min		36°C, 16min

IHC, Immunohistochemistry.

*Heat-induced Antigen Retrieval by 1 mM EDTA in 10 mM Tris buffer (pH8.5).

### DNA Extraction and Microsatellite Instability Scoring

DNA was extracted from paraffin-embedded tissue samples using the QIAamp DNA Tissue Kit (Qiagen, Hilden, Germany) according to the manufacturer’s protocol. MSI analysis was performed on six mononucleotide repeat markers (BAT-25, BAT-26, NR-21, NR-24, NR-27, and MONO-27; **Table 2**) using a Microsatellite Instability Detection Kit (Microread Gene Technology Co., Ltd, Beijing, China). To detect potential specimen contamination, pentanucleotide repeat markers (Penta C and Penta D) and a sex locus marker (amelogenin) were also analyzed for background confirmation. Fluorescently labeled primers were used in the PCR assay, and results were analyzed on an ABI 3130XL gene analyzer with the GeneScan 3.7 analysis software (Applied Biosystems, Foster City, California, USA). Samples were considered MSI-H when two or more of the markers displayed MSI. Samples with MSI at only one marker were considered MSI-L, and those with no MSI were considered MSS.

**Table 2:** Markers and primers of mononucleotide repeat marker analysis.

Marker	Gene	Primer sequence (5’-3’)	GenBank number
BAT-25	c-kit	F: TCGCCTCCAAGAATGTAAGT R: TCTGCATTTTAACTATGGCTC	L04143
Bat-26	MSH2	F: TGACTACTTTTGACTTCAGCC R: AACCATTCAACATTTTTAACCC	U41210
NR-21	SLC7A8	F: GAGTCGCTGGCACAGTTCTA R: CTGGTCACTCGCGTTTAGAA	XM_033393
NR-24	Zinc finger 2	F: GCTGAATTTTACCTCCTGAC R: ATTGTGCCATTGCATTCCAA	X60152
NR-27	Inhibitor of apoptosis protein-1	F: AACCATGCTTGCAAACCACT R: CGATAATACTAGCAATGACC	AF070674 85.1
MONO-27	M4P4K3	F: CAGGGAAATGGTGGGAACCCAG R: GTTGGCCAAGTGAAATTTGATC	AC007684

### Next-Generation Sequencing

Hybrid capture-based targeted next-generation sequencing was performed. Paired tumor and blood tissue DNA samples were extracted from FFPE samples. Barcoded libraries were hybridized to a multiple-gene panel covering whole exons and selected introns of MMR-related genes, including *MLH1*, *MSH2*, *MSH6*, *PMS2*, and *EPCAM*. These libraries were sequenced on an Illumina NextSeq 500 platform and assessed for variants including single nucleotide variants, small insertions and deletions (indels), copy number alterations, and gene fusions/rearrangements. The average sequencing depth for target regions of tumor samples was 8991×, and 97.0% of the average coverage for targeted regions was >1250×.

### Statistical Analysis

Statistical analysis was performed using SPSS 25.0 (SPSS, Chicago, Illinois, USA). Qualitative variables were compared by a chi-square test. Survival curves were prepared according to the Kaplan–Meier method and compared using the log-rank test. *p*-values < 0.05 in two-tailed tests were considered statistically significant.

## Results

### Clinicopathological Characteristics

The median age of the cohort was 49 (range: 24–90). Of the 440 patients enrolled, 376 (85.5%) and 136 (30.9%) had received chemotherapy and radiotherapy, respectively. Breast-conserving surgery was conducted for 52 (11.8%) patients, while the rest underwent radical mastectomy. Basal-like breast cancer was detected in 362 (82.3%) patients with TNBC. Other parameters were also evaluated, such as age, tumor size, P53, Ki-67 index, lymph node metastasis, presence of TILs, and expression of immune checkpoint markers, including PD-1 and PD-L1 (**Table 3**).

**Table 3:** Clinicopathological characteristics of patients.

Clinicopathological criteria	No. of patients	Patients with recurrence	Patients without recurrence
Age at diagnosis			
≤49	212	67	145
>49	228	71	157
Menopausal status			
Pre-menopause	224	73	151
Post-menopause	216	65	151
Histological grade			
I	10	2	8
II	122	40	82
III	308	96	212
Tumor size			
pT1	199	50	149
pT2	219	75	144
pT3	18	12	6
pT4	4	1	3
Nodal status			
negative	247	54	193
1–3 nodes	104	32	72
4–9 nodes	38	16	22
≥10 nodes	51	36	15
TNM Stage			
I	135	25	110
II	211	59	152
III	94	54	40
Surgical procedure			
Mastectomy	388	119	269
Breast conserving	52	19	33
Basal-like phenotype			
Negative	78	25	53
Positive	362	113	249
Ki67 index			
≤14	54	24	30
>14	386	114	272
TILs			
Low	330	121	209
High	110	17	93
Chemotherapy			
Negative	56	17	39
Positive	376	119	257
Unknown	8	2	6
Radiotherapy			
Negative	291	83	208
Positive	136	48	88
Unknown	13	7	6
MSI			
MSI-L	14	6	8
MSS	177	73	104
Unknown	249	59	190
PD-1 (1%)			
Negative	59	36	23
Positive	136	44	92
Unknown	245	58	187
PD-L1 (25%)			
Negative	182	74	108
Positive	13	6	7
Unknown	245	58	187

TNM, Primary Tumor/Regional Lymph Nodes/Distant Metastasis; TILs, tumor infiltrating lymphocytes; MSI, microsatellite instability; MSI-L, low-frequency microsatellite instability; MSS, microsatellite stable; *Ki-67 index threshold of 14% was chosen according to the St. Gallen Consensus 2013.

The median follow-up period was 96 months (range: 2–184 months), with median DFS and OS values of 88 and 96 months, respectively. During the follow-up, there were 138 (31.4%) deaths and 91 (20.7%) cases of recurrence, with 124 (89.9%) and 73 (80.2%) occurring within the first 5 years.

### Mismatch Repair Deficient/High-Frequency Microsatellite Instability Testing

**Figure 1:**
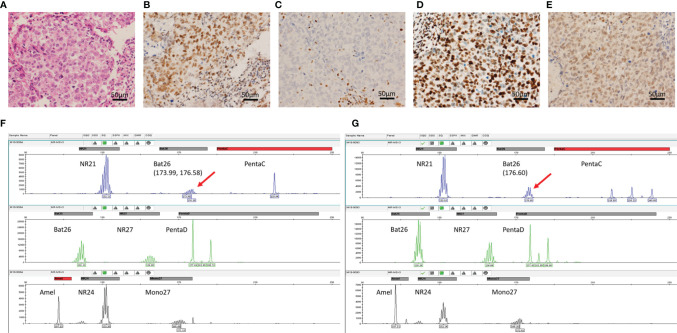
Immunohistochemistry for MMR and MSI PCR analysis. **(A)** Hematoxylin-eosin staining; **(B)** MLH1 staining showed positive nuclei. **(C)** MSH2 staining showed unstained tumor cell nuclei with positively-stained nuclei of stromal cells as normal control. **(D, E)** The high protein expression of PMS2 and MSH-6, respectively. **(F, G)** Graphical maps of the gene loci identified in the DNA sequence of paired tumor and normal tissue samples of the MSI-L case with the mutation loci marked, respectively.

MMR proteins, including MLH1, MSH2, MSH6, and PMS2, were detected in the 440 samples by IHC. MMR protein expression analysis revealed mostly proficient MMR (pMMR) phenotypes. Only one case exhibited loss of MSH2 expression (**Figures 1A–E**), suggestive of a dMMR phenotype. A total of 195 samples of TNBC, including the dMMR sample, were further analyzed for MSI through PCR testing. No MSI-H samples were identified (**Figures 1F, G**), while 14 (7.2%) MSI-L and 181 (92.8%) MSS cases were observed. The dMMR case without MSH2 expression as determined by IHC was categorized as MSI-L by PCR analysis.

**Figure 2:**
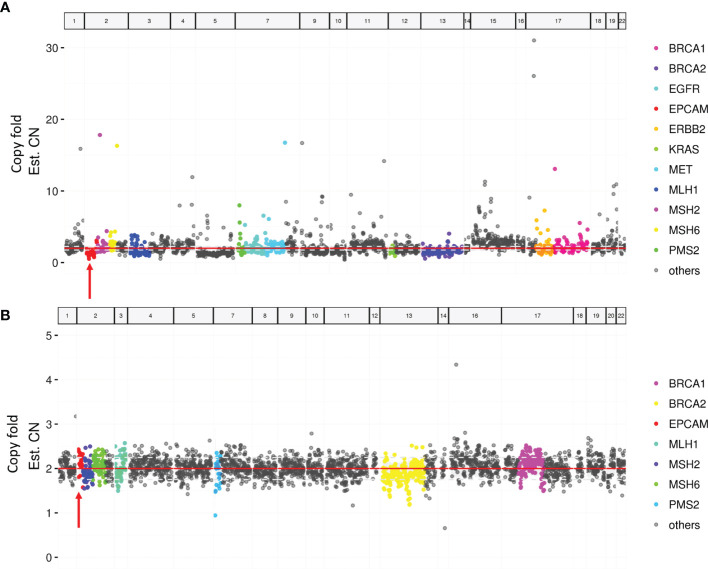
Targeted next-generation sequencing of paired tumor and blood samples. **(A)** A possible *EPCAM* deletion in tumor sample (arrow). **(B)** No *EPCAM* germline deletion in the blood sample (arrow).

Paired tumor and blood samples were collected for MMR gene testing by NGS in the case with MSH2 expression loss. Neither germline nor somatic mutations in MMR genes were detected. However, a possible somatic *EPCAM* copy-number deletion was detected (**Figure 2**).

### Relationship Between Mismatch Repair/Microsatellite Instability Status and Clinicopathological Parameters and Survival in Triple-Negative Breast Cancer

The only dMMR case, which exhibited MSH2 loss, was a 68-year-old woman without any family history of colorectal or endometrial cancer. The patient had high-grade invasive cancer of no special histological type with no lymph node metastasis, positive immunostaining for P53 and basal-like markers (EGFR and CK5/6), and a Ki-67 proliferative index of 35%. This patient, who was the only patient with dMMR, showed no recurrence during this study.

**Figure 3:**
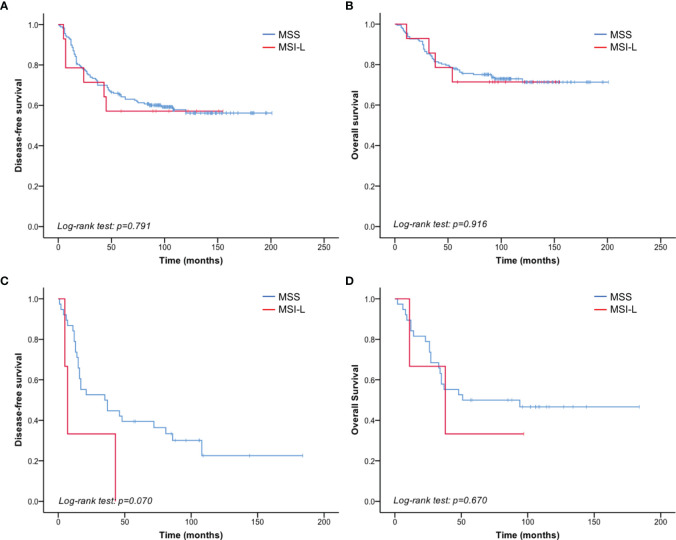
MSI status did not correlate with TNBC prognosis. **(A)** No significant difference in DFS was detected between the MSI-L and MSS patients of all stages. **(B)** No significant difference was detected in OS between the MSI-L and MSS groups of all stages. **(C)** Shorter DFS was observed in MSI-L patients than in MSS patients with stage III breast cancer. **(D)** No significant difference in OS was observed between MSI-L and MSS patients with stage III breast cancer.

Given that no MSI-H patients were identified by PCR testing, we compared the clinicopathological parameters between MSS and MSI-L in 195 patients of known MSI status. There was no significant correlation between MSI status and clinicopathological parameters (**Table 4**). And no significant difference was found in DFS or OS between MSI-L and MSS patients (p = 0.791 and 0.916 of all stages, p=0.073 and 0.671 of stage III, respectively) (**Figure 3**, **Tables 5**, **6**).

**Table 4:** MSI phenotype and pathological parameters.

Pathological Parameters	No. of Patients	MSI phenotype		*p*
		MSS	MSI-L	
Age at diagnosis				0.406
≤49	97	92	6	
>49	98	89	9	
Histological grade				0.546
I	9	8	1	
II	51	49	2	
III	135	124	11	
Tumor size				0.156
pT1	81	78	3	
pT2	106	95	11	
pT3	8	8	0	
Nodal status				0.295
negative	110	101	9	
1–3 nodes	45	43	2	
4–9 nodes	19	19	0	
≥10 nodes	21	18	3	
TNM Stage				0.516
I	52	50	2	
II	101	92	9	
III	42	39	3	
Basal-like				1.000
Negative	43	40	3	
Positive	152	141	11	
Ki67 index*				0.473
≤14	32	31	1	
>14	163	150	13	
TILs				0.753
High	49	45	4	
Low	146	136	10	
Surgery				1.000
Breast-conserving	20	19	1	
Radical mastectomy	175	162	13	
Chemotherapy				1.000
Negative	30	28	2	
Positive	157	146	11	
Radiotherapy				1.000
Negative	149	137	12	
Positive	34	32	2	
P53				1.000
Negative	101	94	7	
Positive	93	86	7	
PD-1				0.129
Negative	59	52	7	
Positive	136	129	7	
PD-L1				0.604
Negative	182	168	14	
Positive	13	13	0	

MSI, microsatellite instability; MSS, microsatellite stable; MSI-L, low-frequency microsatellite instability; TNM, Primary Tumor/Regional Lymph Nodes/Distant Metastasis; TILs, tumor infiltrating lymphocytes; *Ki-67 index threshold of 14% was chosen according to the St. Gallen Consensus 2013.

**Table 5:** Univariate analysis of prognostic value of clinicopathological factors and MSI phenotype of DFS and OS in patients of all stages.

Variable (DFS)	DFS	p	OS	*p*
	HR (95% CI)		HR (95% CI)	
Age at diagnosis		0.374		0.032
≤49			1	
>49			0.539 (0.306–0.949)	
Menopause		0.496		0.913
Pre-menopausal				
Post-menopausal				
Histological grade		0.457		0.945
I				
II				
III				
Tumor size		0.009		0.039
pT1	1		1	
pT2	1.304 (0.813–2.09)		1.742 (0.946–3.207)	
pT3	3.988 (1.646–9.661)		3.784 (1.255–11.409)	
Nodal status		0.000		0.000
negative	1		1	
1–3 nodes	1.851 (1.051–3.261)		1.277 (0.598–2.73)	
4–9 nodes	2.183 (1.035–4.606)		2.625 (1.108–6.217)	
≥10 nodes	8.355 (4.694–14.872)		6.488 (3.303–12.743)	
TNM Stage		0.000		0.000
I	1		1	
II	1.541 (0.817–2.907)		1.428 (0.636–3.209)	
III	4.632 (2.408–8.909)		4.755 (2.113–10.702)	
Basal-like phenotype		0.807		0.169
Negative				
Positive				
Ki67 index*		0.589		0.535
≤14				
>14				
TILs		0.156		0.198
Low				
High				
Surgery		0.265		0.459
Breast-conserving				
Radical mastectomy				
Chemotherapy		0.949		0.234
Negative				
Positive				0.266
Radiotherapy		0.580		
Negative				
Positive				
P53		0.799		0.880
Negative				
Positive				
MSI		0.791		0.916
MSS				
MSI-L				

MSI, microsatellite instability; DFS, Disease-free survival; OS, Overall survival; TNM, Primary Tumor/Regional Lymph Nodes/Distant Metastasis; TILs, tumor infiltrating lymphocytes; MSS, microsatellite stable; MSI-L, low-frequency microsatellite instability.

*Ki-67 index threshold of 14% was chosen according to the St. Gallen Consensus 2013.

**Table 6:** Univariate analysis of prognostic value of clinicopathological factors and MSI phenotype of DFS and OS of patients with stage III.

Variable (DFS)	DFS	*p*	OS	*p*
	HR (95% CI)		HR (95% CI)	
Age at diagnosis		0.991		0.511
≤49				
>49				
Menopause		0.028		0.427
Pre-menopausal	1			
Post-menopausal	2.284 (1.095–4.764)			
Histological grade		0.996		0.600
I				
II				
III				
Tumor size		0.295		0.693
pT1				
pT2				
pT3				
pT4				
Nodal status		0.003		0.155
1–3 nodes	1			
4–9 nodes	1.3 (0.163–10.339)			
≥10 nodes	4.808 (0.636–36.334)			
Basal-like phenotype		0.986		0.616
Negative				
Positive				
Ki67 index*		0.222		0.062
≤14			1	
>14			6.778 (0.909–50.526)	
TIL		0.277		0.330
Low				
High				
Surgery		0.100		0.059
Breast-conserving			1	
Radical mastectomy			0.293 (0.082–1.045)	
Chemotherapy		0.432		0.143
Negative				
Positive				
Radiotherapy		0.037		0.009
Negative	1		1	
Positive	0.435 (0.199–0.951)		0.272 (0.102–0.721)	
P53		0.726		0.525
Negative				
Positive				
MSI		0.073		0.671
MSS	1		1	
MSI-L	2.894 (0.857–9.774)		1.368 (0.319–5.865)	

MSI, microsatellite instability; DFS, Disease-free survival; OS, Overall survival; TNM, Primary Tumor/Regional Lymph Nodes/Distant Metastasis; TILs, tumor infiltrating lymphocytes; MSS, microsatellite stable; MSI-L, low-frequency microsatellite instability.

*Ki-67 index threshold of 14% was chosen according to the St. Gallen Consensus 2013.

## Discussion

Both dMMR and MSI-H have been identified as effective predictors of immunotherapy response (10, 11). The exploration of MSI status as a predictive biomarker has been carried out in a broad range of tumor types. Large-scale analysis showed that MSI-H occurred infrequently (1%–5%) in cancer types not conventionally associated with an MSI-H phenotype (28). Furthermore, MSI testing revealed a larger spectrum of tumors relating to Lynch syndrome compared to previous reports (29). Universal screening for MMR/MSI status is now being recommended in routine oncological care of patients with solid tumors regardless of the cancer’s origin. Consequently, MMR/MSI status in breast cancer has also garnered attention. The purpose of this study was to evaluate the clinical relevance of MMR/MSI status in Chinese women with TNBC in order to determine the frequency of dMMR/MSI-H and its potential for predicting the outcome of immunotherapy.

We demonstrated that the frequency of dMMR and MSI-H was 0.2% (1/440) and 0%, respectively, in a relatively large cohort. To our knowledge, this is the largest TNBC cohort evaluated for dMMR/MSI-H to date. These results were similar to those of a previous report that included a smaller cohort of TNBC patients (30). Our results suggest that dMMR/MSI-H is very rare in sporadic cases of TNBC, although patients with MSI-H may benefit from immune checkpoint inhibitors. The frequency of MMR defects in sporadic breast cancer is reported to be 0% to 20%, while in breast cancer with MMR gene mutations, the frequency of dMMR/MSI-H is reported to be higher, with 65% of patients displaying dMMR and 35% displaying MSI-H (21). Therefore, it may not be feasible to screen for MMR/MSI status in routine clinical tests unless there is proof of Lynch syndrome-related cancer, such as family history or the presence of multiple tumors (31). Evaluation of TILs in the TNBC microenvironment might be more useful for predicting the efficiency of immunotherapy (32). Using a relatively large cohort, our study conclusively showed the extremely rareness of dMMR/MSI-H in TNBC, which indicated that it might be necessary to identify other biomarkers for predicting immunotherapy outcomes in TNBC, such as TILs and immune gene evaluation. However, in view of the extremely low frequency of dMMR/MSI-H, whether MSI/MMR status is an effective indicator of immunotherapy response or prognosis in patients with TNBC needs further clarification.

In dMMR tumors, MSH2 protein loss, traditionally evaluated by immunochemical staining, generally occurs concurrently with loss of MSH6 protein expression. However, one patient in our cohort exhibited an abnormal staining pattern, showing an isolated loss of MSH2 with intact MSH6 expression. This discordance in IHC results could not be ascribed to the variable reactivity or subjective interpretations, given the robust internal control (intervening stroma) used in the staining procedure. Similar staining patterns (retained MSH6 expression with the absence of MSH2), which were attributed to germline or somatic MSH2 mutations, have been previously reported in colorectal cancer (33). To confirm the MSI status, PCR was performed. Intriguingly, PCR showed that the dMMR tumor was MSI-L, indicating an inconsistency between the results of the MMR IHC staining and the MSI test. This unusual inconsistency has been rarely reported in cancers that are not typical Lynch syndrome-related tumors, including adrenocortical carcinoma, peritoneal mesothelioma, pancreatic acinar cell carcinoma, and pancreatic neuroendocrine tumors (34). One potential explanation is that the accumulation of detectable MSI was a secondary event that only occurred at a later stage, after the dysfunction of MMR proteins. To further clarify the genetic mechanisms underlying the MSH2 protein loss in this case, we conducted NGS-based testing. Results from NGS testing revealed a possible *EPCAM* copy-number deletion in the tumor tissue. It has been documented that 3’ *EPCAM* deletion causes transcriptional read-through of the mutated *EPCAM* allele, resulting in epigenetic inactivation and silencing of its neighboring gene *MSH2* (35).

dMMR/MSI is useful for predicting treatment outcomes for some malignancies, including colon cancer (15). Previous studies have evaluated dMMR/MSI status in breast cancer, but the prognostic significance was inconsistent. One study on 248 patients with breast cancer demonstrated that MSI-H has no significant impact on patient survival or PD-L1 expression (30). In contrast, some studies have reported the prognostic value of dMMR/MSI-H status in breast cancer (20, 22–24). In our cohort of TNBC patients, only one patient could be characterized as dMMR based on the lack of MSH2 expression. This patient did not exhibit recurrence and/or metastasis until the final follow-up. PCR and NGS only identified a small number of MSI-L cases. Unlike MSI-H tumors, MSI-L tumors appear to arise *via* the chromosomal instability pathway (36). Few studies have described the prognostic value of MSI-L. Therefore, we further analyzed the prognostic significance of MSI-L and its relationship with clinicopathological characteristics in TNBC. Our retrospective study revealed that patients with an MSI-L phenotype had relatively poorer survival than those with an MSS phenotype. Of the patients with stage III cancer, MSI-L patients had a median DFS of 7 months, while MSS patients had a median DFS of 35 months. However, the difference in DFS was not significant (p = 0.073). Similar results have been observed in patients with colon cancer (37). No significant relationship was found for MSI-L and age, tumor size, grade, Ki-67 index, P53, PD-1/PD-L1 expression, and the number of TILs. There have been new advances in increasing the efficacy of immunotherapy in colorectal cancers that are MMR-proficient and characterized as MSI-L (38). However, albeit the lack of significant correlation between MSI-L phenotype and clinicopathological characteristics in our study, we should be aware of the limitations imposed by the small numbers of cases with MSI-L, and further studies are needed to validate the findings in a larger cohort.

In conclusion, the incidence of dMMR/MSI-H is extremely low in patients with TNBC. Moreover, MMR/MSI status was not associated with PD-1/PD-L1 expression and showed little prognostic significance in TNBC. Further studies are required to explore biomarkers with a predictive capacity for immunotherapy outcomes in patients with TNBC.

## Data Availability Statement

The sequencing data has been deposited into BioProject (accession: PRJNA639238).

## Ethics Statement

The studies involving human participants were reviewed and approved by the Peking Union Medical College Hospital Institutional Review Board (PUMCH IRB). The patients/participants provided their written informed consent to participate in this study.

## Author Contributions

X-yR conceptualized the study, developed the methodology, performed the formal analysis, and wrote, edited, and reviewed the manuscript. YS conceptualized the study, developed the methodology, conducted the investigation, was in charge of the data curation, and wrote the manuscript. L-yC developed the methodology, conducted the investigation, and provided the resources. J-yP developed the methodology, conducted the investigation, and provided the resources. S-jS developed the methodology and reviewed the manuscript. M-mS developed the methodology and reviewed the manuscript. Z-yL conceptualized the study, developed the methodology, reviewed the manuscript, acquired the funding, and supervised the study. QS conceptualized the study, reviewed the manuscript, was in charge of the project administration, and supervised the study. H-wW conceptualized the study, developed the methodology, reviewed the manuscript, and was in charge of the project administration. All authors contributed to the article and approved the submitted version.

## Funding

This study was funded by CAMS Central Public Welfare Scientific Research Institute Basal Research Funds Clinical and Translational Medicine Research Program (2019XK320045) and CAMS Initiative for Innovative Medicine (2016-I2M-1-002). The funding source took no part in the design or conduct of the study.

## Conflict of Interest

M-mS was employed by the company Beijing Microread Genetics Co., Ltd.

The remaining authors declare that the research was conducted in the absence of any commercial or financial relationships that could be construed as a potential conflict of interest.
